# Medical Assistance in Dying in Quebec: A Continuum Between Teams’ Accountability and Interdisciplinary Support Groups’ Assumption of Responsibility

**DOI:** 10.3389/ijph.2024.1607407

**Published:** 2024-08-29

**Authors:** Catherine Perron, Eric Racine, Marie-Eve Bouthillier

**Affiliations:** ^1^ Centre Integre de Sante et de Services Sociaux de Laval, Laval, QC, Canada; ^2^ Department of Medicine, Montreal University, Montreal, QC, Canada; ^3^ Montreal Clinical Research Institute (IRCM), Montréal, QC, Canada; ^4^ Department of Medicine, McGill University, Montreal, QC, Canada

**Keywords:** medical assistance in dying, coordination, support structures, values, pragmatic ethics

## Abstract

**Objectives:**

In the province of Quebec, Canada, interdisciplinary support groups (ISGs) are mandated to support those who are involved in the clinical, administrative, legal and ethical aspects of medical assistance in dying (MAiD). This article presents the results of a mixed-method, multi-phase study carried out in 2021 on ISGs with the aim to describe current ISG practices, critically analyze them and make recommendations on promising practices for provincial implementation.

**Method:**

Semi-structured interviews (42) and focus groups (7) with coordinators of 24 ISGs were used to identify promising practices and confirm their utility with participants.

**Results:**

We have distributed the ISGs along what we coined an “ISG continuum.” Between teams’ accountability (decentralization) and ISGs’ assumption of responsibility for MAiD requests (centralization), a middle ground approach, focused on the value of support, should be favored.

**Conclusion:**

The structuring of ISGs and their practices is intimately linked to their values. Harmonization of ISGs and their practices, while considering their specific values and contexts, can contribute to the equity and quality of services intended for those who request MAiD and those who support them.

## Introduction

The practices of euthanasia and assisted suicide have undergone unprecedented international expansion over the past 20 years. In Quebec, the practice has been legalized since December 2015 under the term “medical assistance in dying” (MAiD). It is defined as “care consisting in the administration by a competent professional [physician or nurse practitioner] of medications or substances to a patient, at the patient’s request, in order to relieve their suffering by hastening death.” [1]. Regulatory bodies confirm the growing number of MAiD requests that Quebec health and social services professionals must respond to. In the last year, Quebec experienced a growth curve unmatched nationally and internationally [2]. Deaths by MAiD have reached a provincial average of 6.8%, while some regions have approached 10% [3]. For each request, physicians, nurses, and pharmacists are involved. Social workers, spiritual care workers, ethicists, administrators, and other partners may also participate in MAiD trajectory if need be. In the province, interdisciplinary support groups (ISGs) are mandated to support these people in the clinical, administrative, legal, and ethical practices of MAiD.

When ISGs were first implemented, ministerial directives gave healthcare institutions a great deal of freedom in implementing these MAiD support structures [4]. More than 5 years after their implementation, significant variability is observed in the constitution, roles and functioning of ISGs. Based on available data regarding the practice of MAiD in Quebec, we hypothesize that this variability may have an impact on the type of support provided and it’s availability to healthcare providers, as well as access to care and services for patients. Indeed, reports from the Commission on End-of-Life Care (the body responsible for examining the conformity of end-of-life care in Quebec) confirm an inter-regional variability in administered and non-administered MAiD requests. In light of the “variability of the sources declared” by the establishments, the commission calls for caution in interpreting the data [5, 6]. Furthermore, exchanges between members of the ISG’ Community of Practice point to inter-regional and inter-institutional disparities, suggesting that this has an impact on patients, families and healthcare teams. Other issues are also likely to emerge from this variability, notably the quality of the act, public confidence, the credibility of institutions and the transparency and integrity of the MAiD process. In the interests of those who request MAiD and in those who support its provision, it is essential to study ISG structures, their development, relevance, values, and vision. We conducted a mixed-method, multi-phase research project to describe current ISG practices, critically analyzed them and made recommendations in the form of promising practices for provincial implementation. In this article, we present the results and analysis as they pertain to the coordination of MAiD requests by Quebec’s ISGs. Inspired by John Dewey’s pragmatic ethics, we analyzed the values espoused by ISGs. More specifically, we flesh out the position of the ISGs on the practices of centralizing and decentralizing MAiD requests in their organization. We demonstrate how social, collective, and institutional values have shaped the development of ISGs, and how these structures in turn shaped the support offered to healthcare providers and the care offered to MAiD requestors.

### Overview of International Support Structures for Medical Assistance in Dying

With the development of MAiD in several provinces, states and countries around the world, various structures have been created to support caregivers involved in this practice. The first structures that have been researched were those in the Netherlands and Belgium [7–9]. In these countries, support structures such as SCEN or LEIF are not defined as “central coordination centres for MAiD requests,” but rather as structures offering support to MAiD evaluators, particularly in the search for a second independent evaluator. On the other hand, the Expertisecentrum Euthanasie in the Netherlands [10] handles the entire MAiD process, but solely for clinically complex situations in which attending physicians do not wish to become involved as assessors. Conversely, in Canada, some structures are mandated to receive MAiD requests for an entire province or region, and coordinate the whole trajectory and process.

Researchers who have studied support structures in the country are generally in favor of a centralized model for the coordination of MAiD requests. For example, Wiebe et al. [11] recommend that care coordination centres be established in each province or health region. The authors present the centralized model as a practice worth adopting, because it avoids placing the entire burden of responsibility of the MAiD process solely on the shoulders of providers. The aim of these centralized coordination centres is to reduce and share the burden – administrative, professional, moral, and emotional – on all those involved in the MAiD process. It also has the potential of ensuring better transmission of information to patients. According to the authors, the mandate of coordination centres should consist of supporting patients and clinicians, including those who are reluctant to become involved in practice.

Coordination centres in British Columbia and Alberta are responsible for informing patients about MAiD and referring them to potential evaluators and providers [11, 12]. Oczkowski’s et al. [13] claim that MAiD coordination is central to the quality of care for patients and families given that it encompasses the entire MAiD trajectory, from the request through to the act itself and in the post-death support of relatives.

The centralization of MAiD support structures and the creation of “MAiD coordinator” positions are considered in the Canadian literature as practices that can improve the quality of MAiD provision. Prior to the launch of our research, no empirical data existed on the specific models of Quebec’s ISGs. However, we knew that their initial mandate, which was drawn up by the Ministry of Health and Social Services, did not include any specific responsibility for informing the population or referring applicants to medical evaluators [4]. Nor did their mandate include providing direct support to patients or their relatives. Instead, it was proposed that the structures be formed *by* and *for* the stakeholders concerned by the practice. We also knew that each healthcare institution was invited to set up such a support structure, which would amount to nearly thirty ISGs [14].

## Methods

Three methods were chosen to meet our objectives of describing, analyzing and valuing ISG practices. In the first phase, all those involved in MAiD in Quebec (e.g., physicians, nurses, social workers, administrators, MAiD coordinators) were invited to complete a mixed online questionnaire. The results and analyses from this phase have been previously published [14]. For the second and third phases (reported here), we conducted semi-structured interviews (N = 42) and focus groups (N = 7) with the coordinators (N = 42) of 24 ISGs. NVivo-12 software was used for qualitative thematic analysis. A codebook was initially developed based on the literature related to the research topic. In doing so, we aimed to circumscribe the object of analysis and to facilitate data reduction [15]. The first interviews were conducted using emergent coding to highlight themes emerging from the participants’ discourse, but also to refine the initial codebook throughout the coding process [16]. The key themes for this analysis were related to: a) ISGs composition; b) collaboration with and between MAiD regulatory bodies in Quebec; c) ISGs activities; d) ISGs functioning and e) ISGs values and positioning (reported here). Participant quotes were anonymized, and participants were designated by their gender letter (M; W) and a number (e.g., M1, W2), while ISGs were assigned a letter (e.g., A, B). Content from the different themes is summarized narratively and using tables or figures. Salient examples are cited to illustrate important aspects of the themes.

Our analysis was based on John Dewey’s pragmatic ethics and his theory of valuation [17, 18]. According to this theory, individuals affected by a particular problem are seen as those best suited to contribute to its resolution [19]. In fact, Dewey proposes that problematic situations should be analyzed using a bottom-up approach. That is, starting from the needs expressed by the agents affected by a given problem. These needs, in turn, serve as a springboard towards their “ends-in-view,” i.e., the anticipated goals and consequences that ultimately guide the possible course of action chosen [20]. In the context of great variability among ISG practices, pragmatic ethics suggests that the specific needs of ISGs will drive their aspirations, structures, and practices. The context in which practices are applied is therefore of prime importance. To qualify as “ethical,” a practice must be subjected to a contextual analysis, evaluated for its potential contribution to human development [21], and must consider the temporal, material and human components in which it is applied [22].

Analysis of our data enabled us to classify the ISGs according to their respective contexts and resources. We were also able to identify the values espoused by ISGs included in our study. The classification of their values then enabled us to distribute them along what we called the “ISG continuum.” This continuum was presented to the participants, who were then asked to situate where they felt promising ISG practice lay along this continuum.

## Results

### ISGs as Reception and Coordination Centres for MAiD Requests

Two main types of ISGs emerged from the research based on their internal structure and functioning for receiving and coordinating MAiD requests. Fifty-eight percent of the sample (n = 14) consisted of ISGs that had a centralised processing centre for treating MAiD requests, while 42% (n = 10) did not have such a centre.

ISG coordinators working in a ISG following a centralised model put forward several arguments in favor of their internal structure such as the duty of accountability to patients and respect for their rights. More specifically, they reported that the centralised centre acts as a watchful eye by limiting the number of MAiD requests that may go unanswered while ensuring that urgent requests are treated in a timely fashion. Moreover, this model trains healthcare teams, raises awareness regarding MAiD provision, identifies team support needs and prevents resource exhaustion through fair and strategic distribution of MAiD requests. Lastly, the centralised structure enables ISGs to better anticipate the need for MAiD assessment resources and facilitates reporting to Quebec’s regulatory body, the Commission on End-of-Life Care:

We act as a kind of watchdog … it already enables us to anticipate services and organize care, to coordinate our activities around these future requests. (W3)

I know how many requests we have, how many pending requests. I know which doctors are going to do it, so if they get too many requests, I ask someone else to do it, and you know, I have a global view of all the requests coming in. So, I think that’s an advantage … In terms of accountability, it’s also an advantage. How many MAiD requests, how many continuous palliative sedations. It takes quite a long time [to report], but our information is in the same place, so it’s easier. (M6)

ISG coordinators from the decentralised model also evoked arguments in support of their structure. First, they pointed to the lack of resources required to set up such a centre. They also highlighted the complexity and burden of the task, the challenge of responding promptly to patient requests and the difficulties involved in recruiting physicians. But above all, they emphasized the risk of disempowering healthcare teams, particularly doctors:

The doctors on the ISG were absolutely against this way [of centralizing requests] because it actually takes responsibility away from the doctors; they send it to someone else. (W21)

I wouldn’t want it to become too easy, and for them to lose responsibility for their role as professionals in this area (M5).

A centre means that we’re passing our responsibility on to someone else, and the idea is precisely the therapeutic patient-team-doctor relationship. The centre gives us peace of mind that someone else will take care of it, but it also takes away responsibility. (W32)

The discussion groups enabled participants to exchange views on the various existing ISG models. We asked participants whether the introduction of a one-stop shop for receiving and coordinating MAiD requests was a promising practice for their organization. It is interesting to note that, despite the participants’ inclination to defend their model in the discussions, most of them answered this question in the affirmative. However, several said that a lack of resources prevented the implementation of centralised structure, as this participant testifies:

I’m against a one-stop shop for medical aid in dying, if there aren’t additional resources, budget, funding, and everything else that goes with it, because otherwise, it’s unthinkable with the number of requests there are! (W17)

### ISG Values: Between Teams’ Accountability and ISGs’ Assumption of Responsibility

The values evoked by all the participants have been grouped together in Table 1 into what one of them described as their “core values” (H11). These values form the foundation on which ISGs members build.

**TABLE 1:** Common core values in interdisciplinary support groups (Mixed Research on Interdisciplinary Support Groups for Medical Assistance in Dying: Analysis of Promising Practices and Recommendations for their Implementation, Quebec Canada, 2021).

Respect Quality of life Dignity Compassion Support Solidarity Commitment	Listening Sharing Meaning/depth Humanism Empathy Caring Professionalism Fairness and justice

**FIGURE 1:**
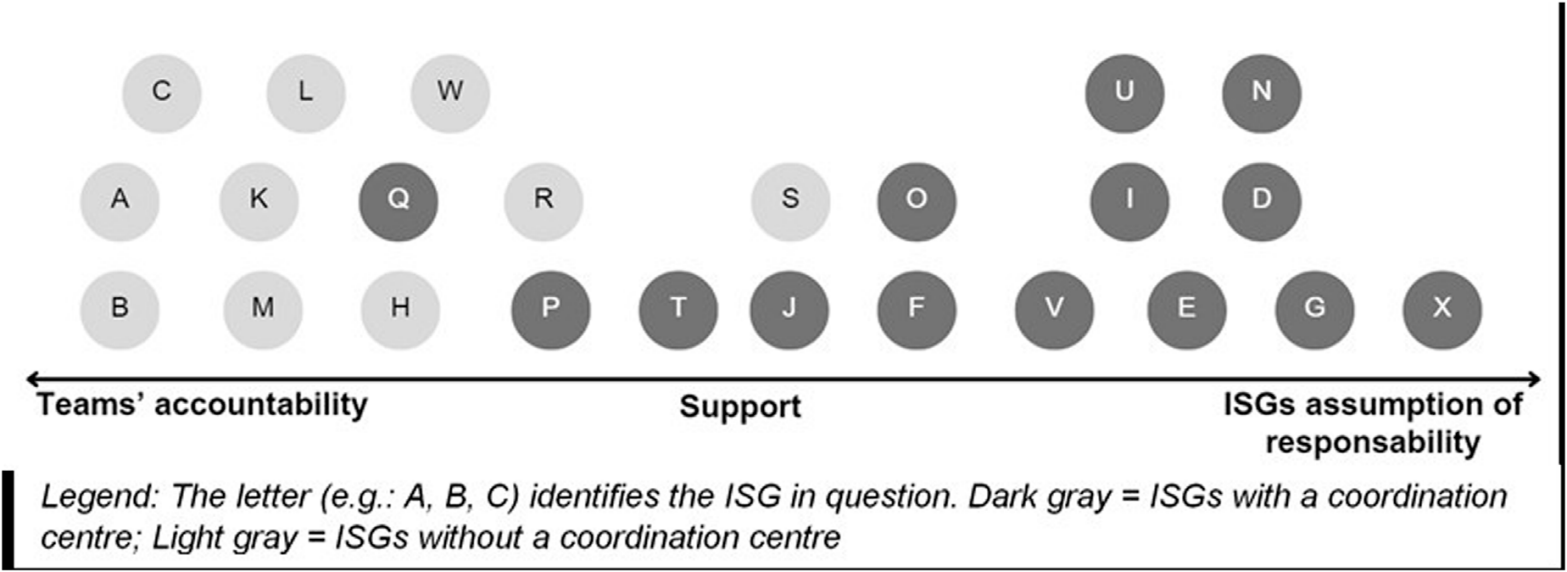
Interdisciplinary support groups continuum (Mixed Research on Interdisciplinary Support Groups for Medical Assistance in Dying: Analysis of Promising Practices and Recommendations for their Implementation, Quebec Canada, 2021).

In addition to these shared values, the ISGs were distinguished by the values they uphold. Based on these distinctive values, we were able to distribute the ISGs along what we have termed the “ISG continuum” (Figure 1). On the left are ISGs that promote the accountability of healthcare teams in the management of MAiD requests. On the right, we find the ISGs that defend the ISGs’ assumption of responsibility regarding MAiD requests. In the center are the ISGs that emphasize team support. According to our analysis, the ISGs positioned on the assumption of responsibility side all have a centre for receiving and coordinating requests. However, there are also ISGs at the center of the continuum with a centralised structure. Meanwhile, none of the ISGs on the far left of the continuum had a centralised centre. In Figure 1, ISGs with a coordination centre are shown in dark gray, while ISGs without a coordination centre are shown in light gray.

The coordinators whose ISG is positioned on the side of accountability had a firm stance towards physicians and healthcare teams. Their arguments were based on medical responsibility and access to MAiD:

Whether the patient is anywhere, in any institution, at home, in a nursing home, name it, it will be the doctor in charge of that patient who will be responsible … He remains responsible for medical assistance in dying. That’s something we really insisted on. If he’s not comfortable with it, it’s HIS responsibility to find a colleague to do it … It’s one form of care among many, so why should we take more responsibility for it than for others? (W21)

The discourse of participants whose ISG is positioned on the assumption of responsibility side referred to the duty of non-abandonment of healthcare providers, to their obligations towards patients and to the protection of their rights:

Even the units that understand the process well, it’s us who find the doctor. It’s not really the vision that it's going to be the attending physician who’s going to do it. We have a team of doctors who will provide the care. (W27)

You know, our ISG is mainly about finding a doctor. It’s the ISG’s responsibility to find the doctors. (W37)

Two main issues emerged from this model: the overburdening of the ISG and the disempowerment of doctors vis-à-vis patients:

The trap of our model is to take everything and take too much … That’s for sure, doctors unload on us, and that’s the downside (W34).

ISGs at the center of the continuum had a discourse that straddled the two poles. They were concerned with “not to be too directive” towards healthcare providers, and “not to take charge” of MAiD requests. They use terms like “support,” “tools,” “education” and “ownership” to describe their roles:

When we take the time, when doctors see that we’re there to assist and support them at every stage, that the process is well organized and structured, they’ll often say “Ah ok, I’ll do it” (M7).

We don’t want to be a team that takes charge, but we really want to be people who support the teams who are faced with this reality … We find there are pitfalls when you centralize a team. I’m always going to stay, until the request is taken care of and I know it’s going to happen. I’ll network, but I’ll never do “instead of.” Never. (M3)

In short, there are advantages and disadvantages to both models, and the transition from one to the other involves a number of issues, including the empowerment of healthcare providers, their wellbeing, the enhancement of their knowledge, the personalization of the care offered to patients, and finally, the realism of the chosen model for institutions spread over vast territories.

### Support as a Promising ISG Practice

When we asked participants where they saw the promising practice of ISGs in relation to MAiD coordination, a strong majority placed them at the center of the continuum. No participant positioned promising practice in the ISG’s complete handling of MAiD requests. On the other hand, one participant illustrated the trajectory that an entity – ISG or person – can take on the ISG continuum:

You made me react when you showed your ISG continuum. Because I’ve seen myself all over the place, depending on the years and my emotional charge. So, on the one hand, you want to make doctors or healthcare teams accountable, and then, all of a sudden, sooner or later, I end up on the right-hand corner with the burden that I don’t want it to fail … I want to meet patients’ needs. I walk from left to right, sometimes, as a respondent at that level. (M21)

At the end of the analysis, we understood that the context can make people swing from left to right on the ISG continuum. Personal elements such as emotional charge seem to have an impact on this positioning, as well as the timing of the MAiD, the culture of the institution, the values of the ISGs and healthcare providers, as well as the types of resources and their availability. According to the participants, ISGs must aspire, as far as possible, to regain the center of the continuum to fulfill the ISG’s mandate to support teams. This is the most promising position: “You talked about the middle ground, and I think that’s what we should be aiming for – it’s the doctor’s responsibility but supported by a team. I think that’s what we’re going to have to aim for.” (M4).

## Discussion

Broken down into three phases, the overall aim of our research was to describe current ISG practices, critically analyze them and identify practices deemed to be promising. To achieve this, we drew on John Dewey’s pragmatic ethics and deployed a mixed, multi-phase methodology (1. Questionnaire; 2. Semi-structured interviews; 3. Focus groups). Our study highlights two main ISG models: ISGs *with* and *without* a centralised processing centre for MAiD requests. Furthermore, although they share common values, ISGs have been built on values that also distinguish them, enabling us to distribute them along what we have termed the “ISG continuum,” ranging from teams’ accountability to ISGs’ assumption of responsibility, via ISG support for healthcare teams.

While some authors, such as Wiebe et al. [11] and Oczkowski et al. [13], argue that MAiD coordination centres reduce the burden on providers, our research highlights the risks of disempowering teams by centralizing and taking over requests through the ISG. Our research highlights that available resources can also limit the benefits of ISGs assumption of responsibility. ISGs cannot simply choose to position themselves as a coordination centre because this is a “promising practice.” They have to take into account their specific context where values and available resources are at play. Between the two poles of the continuum, our data shows that a middle-ground based on the value of support is favored.

Admittedly, there are certain circumstances in which the role of empowering teams (teams’ accountability) or having the ISG take charge of MAiDs (ISGs’ assumption of responsibility) are best suited. Similarly, the exclusion or exclusivity of one model over the other hinders the ethical and pragmatic nature of the approach, since it requires agents to ignore the values involved and the contexts of action. By considering the plurality of perspectives, probing the intention and needs of stakeholders, and considering available resources, pragmatic ethics guides ISGs towards the preferred posture between teams’ accountability, supporting and ISGs’ assumption of responsibility for MAiD requests.

Drawing on Dewey’s [18] theory of valuation, we propose that the values promoted by ISGs, between teams’ accountability and ISGs’ assumption of responsibility, have shaped their structure – their constitution and practices – and that the structures themselves have a direct impact on the support available to caregivers.

**FIGURE 2:**
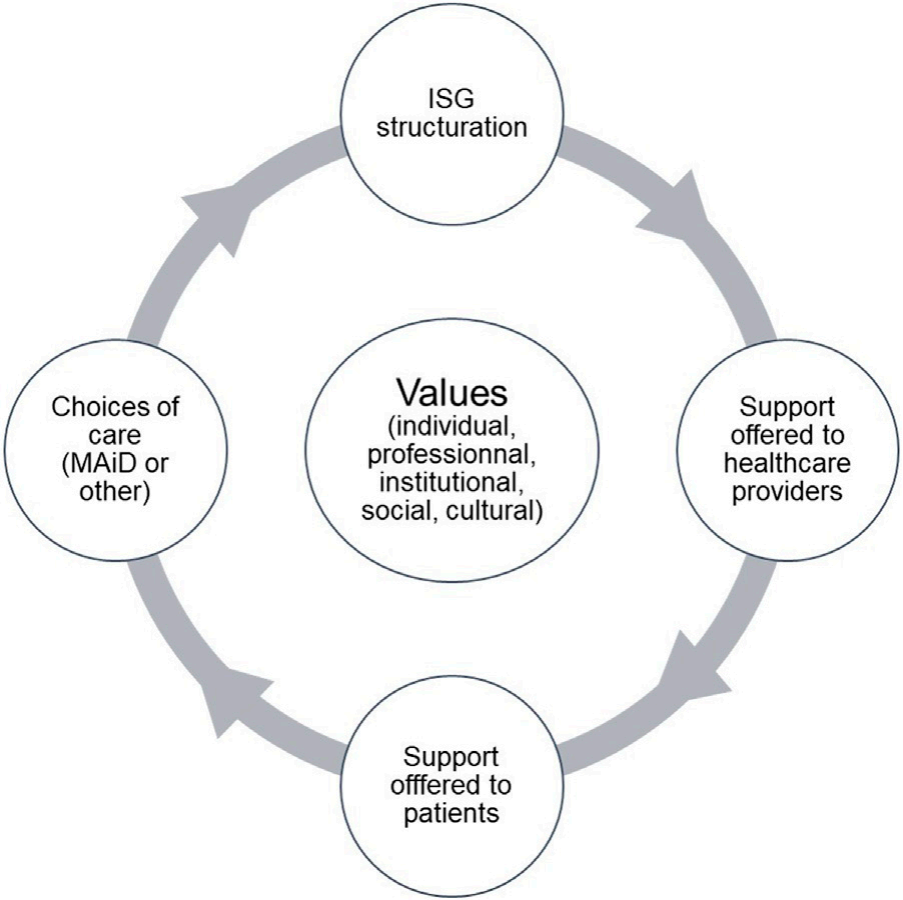
Relationship between values, interdisciplinary support groups structuring, stakeholder support and patient care (Mixed Research on Interdisciplinary Support Groups for Medical Assistance in Dying: Analysis of Promising Practices and Recommendations for their Implementation, Quebec Canada, 2021).

Access to, and the quality of, this support has a direct impact on the information and support provided to patients, enabling them to make end-of-life care choices based on their own values. Finally, these individual values are embedded in, and feed into, the assemblage of social, cultural, collective and institutional values of each healthcare facility. Figure 2 illustrates the relationship that emerges from our analysis.

This illustration gives us a better understanding of how ISGs are structured across Quebec and goes some way to clarify their variability. Currently, the Commission on End-of-Life Care and the Government of Quebec are attempting to understand regional disparities in the practice of MAiD, as MAiD death rates vary across the province between 4.5% and 10.5% [3]. In fact, recent Canadian literature demonstrates that it is easier for patients to obtain MAiD in urban centers than in the regions [11, 23, 24]. Yet, this is not what the Commission on End-of-Life Care’s statistics show [3, 5, 6]. Nor is it what we observed in our research [25]. Indeed, some regional ISGs stand out for their responsiveness in handling MAiD requests and supporting stakeholders in practice. Cross-referencing our data with that of the Commission on End-of-Life Care leads to the hypothesis that in Quebec, patient access to and choice of MAiD may also be impacted by the ISG coordination model, which in turn is impacted by the values espoused by ISGs and the organizational culture of institutions. Future research should thus pay particular attention to the relationships between the values promoted by organizations, ISGs, their members, patients, and the public.

### Limitations

Certain limitations may reduce the scope of our method, analysis, and results. First, the low number of scientific articles written on support structures for MAiD presented a challenge in formulating the research problem. This issue forced us to go beyond scientific writings to explore gray literature, government reports and the sites of equivalent support structures around the world. As our research is devoted to ISGs, it is mainly administrators and caregivers who constitute our sample. Relatives and patients-partners were poorly represented, while patients and relatives themselves were not. Likewise, pediatric, and psychiatric settings were underrepresented in our research. With the potential expansion of MAiD to these populations, new research should be carried out on their specificities and on the ISG services from which they could benefit. In addition, the study reported in this article was carried out in Quebec, where only MAiD in the form of euthanasia is permitted. In jurisdictions where assisted suicide is practiced, the needs of physicians and other professionals may differ. In particular, the fact that physicians are generally not present at the moment of death may affect their needs and responsibilities.

### Conclusion

In Quebec, MAiD has spread in leaps and bounds, to the point where this jurisdiction is the place in the world where the prevalence of the practice has increased most rapidly. ISGs are the main support mechanism for those involved in MAiD, but their existence, mandate and practices remain poorly understood. Our research aimed to understand the variability of ISG practices, their composition, roles, and functioning. Our objective was to describe current ISG practices, critically analyze them and identify practices deemed to be promising. We paid particular attention to the values and practices associated with the coordination of MAiD requests by the ISGs.

The ISGs were distributed along a “continuum of ISG.” Between teams’ accountability and ISGs’ assumption of responsibility, the supportive approach was identified by our participants as the posture to be favored. An analysis carried out from the perspective of pragmatic ethics enables to glimpse the complementarity of the models and transcend their opposition. The contexts and values of the ISGs, their members and their organizations guide their structuring and practices.

At the end of the research, we can relate the variability of ISG practices to their espoused values. This observation leads us to suggest that these variabilities may have led to real issues in terms of access and quality of support for people affected by MAiD across the province. Disparities in ISG operations, practices and service provision may have contributed to healthcare provider confusion and misunderstanding regarding these MAiD support structures. These elements relating to ISGs and the organization of MAiD in Quebec will be taken up and studied by the Interdisciplinary Research Consortium on MAiD [of which two of the authors are a part (Bouthillier and Perron)], a team mandated to better understand the use of MAiD in Quebec. Faced with the many challenges posed by the sharp rise in requests for MAiD in Quebec, and with the forthcoming broadening of eligibility criteria to people with neurocognitive disorders through advance requests [1], harmonization of the ISGs and their practices, taking into account their contextual and regional specificities, should contribute to the equity and quality of services intended for people requesting MAiD and those who support them.
