# Corrigendum: Medical Assistance in Dying in Quebec: A Continuum Between Teams’ Accountability and Interdisciplinary Support Groups’ Assumption of Responsibility

**DOI:** 10.3389/ijph.2024.1607992

**Published:** 2024-10-15

**Authors:** Catherine Perron, Eric Racine, Marie-Eve Bouthillier

**Affiliations:** ^1^ Centre Integre de Sante et de Services Sociaux de Laval, Laval, QC, Canada; ^2^ Department of Medicine, Montreal University, Montreal, QC, Canada; ^3^ Montreal Clinical Research Institute (IRCM), Montréal, QC, Canada; ^4^ Department of Medicine, McGill University, Montreal, QC, Canada

**Keywords:** medical assistance in dying, coordination, support structures, values, pragmatic ethics

In the published article, there was an error in the **Funding** statement. The original statement should have included “the Research Institutes of Canada (EG4 179448)” The correct **Funding** statement appears below.

